# The Impact of Silent Liver Disease on Hospital Length of Stay Following Isolated Coronary Artery Bypass Grafting Surgery

**DOI:** 10.3390/jcm13123397

**Published:** 2024-06-10

**Authors:** Giancarlo Suffredini, Lan Le, Seoho Lee, Wei Dong Gao, Michael P. Robich, Hamza Aziz, Ahmet Kilic, Jennifer S. Lawton, Kristin Voegtline, Sarah Olson, Charles Hugh Brown, Joao A. C. Lima, Samarjit Das, Jeffrey M. Dodd-o

**Affiliations:** 1 Department of Anesthesiology and Critical Care Medicine, Division of Cardiac Anesthesia, Johns Hopkins University School of Medicine, Baltimore, MD 21205, USA; lan.le@gwmail.gwu.edu (L.L.); wgao3@jhmi.edu (W.D.G.); cbrownv@jhmi.edu (C.H.B.); jdoddo@jhmi.edu (J.M.D.); 2 Department of Anesthesiology and Critical Care Medicine, Johns Hopkins University School of Medicine, Baltimore, MD 21205, USA; slee577@jhu.edu (S.L.); sdas11@jhmi.edu (S.D.); 3 Department of Surgery, Division of Cardiac Surgery, Johns Hopkins University School of Medicine, Baltimore, MD 21205, USA; mrobich4@jhu.edu (M.P.R.); haziz2@jhmi.edu (H.A.); akilic2@jhmi.edu (A.K.); jlawton4@jhmi.edu (J.S.L.); 4 Biostatistics, Epidemiology, and Data Management Core, Johns Hopkins University, Baltimore, MD 21205, USA; kvoegtl1@jhu.edu (K.V.); solson16@jhmi.edu (S.O.); 5 Department of Medicine, Division of Cardiology, Johns Hopkins University School of Medicine, Baltimore, MD 21205, USA; jlima@jhmi.edu

**Keywords:** shear wave elastography, liver stiffness, cardiac surgery, coronary artery bypass graft, risk stratification, perioperative, outcomes

## Abstract

**Objectives:** Risk assessment models for cardiac surgery do not distinguish between degrees of liver dysfunction. We have previously shown that preoperative liver stiffness is associated with hospital length of stay following cardiac surgery. The authors hypothesized that a liver stiffness measurement (LSM) ≥ 9.5 kPa would rule out a short hospital length of stay (LOS < 6 days) following isolated coronary artery bypass grafting (CABG) surgery. **Methods:** A prospective observational study of one hundred sixty-four adult patients undergoing non-emergent isolated CABG surgery at a single university hospital center. Preoperative liver stiffness measured by ultrasound elastography was obtained for each participant. Multivariate logistic regression models were used to assess the adjusted relationship between LSM and a short hospital stay. **Results:** We performed multivariate logistic regression models using short hospital LOS (<6 days) as the dependent variable. Independent variables included LSM (<9.5 kPa, ≥9.5 kPa), age, sex, STS predicted morbidity and mortality, and baseline hemoglobin. After adjusting for included variables, LSM ≥ 9.5 kPa was associated with lower odds of early discharge as compared to LSM < 9.5 kPa (OR: 0.22, 95% CI: 0.06–0.84, *p* = 0.03). The ROC curve and resulting AUC of 0.76 (95% CI: 0.68–0.83) suggest the final multivariate model provides good discriminatory performance when predicting early discharge. **Conclusions:** A preoperative LSM ≥ 9.5 kPa ruled out a short length of stay in nearly 80% of patients when compared to patients with a LSM < 9.5 kPa. Preoperative liver stiffness may be a useful metric to incorporate into preoperative risk stratification.

## 1. Introduction

Liver cirrhosis impacts outcomes following cardiac surgery [1,2]. Human [3] and animal [4,5] studies suggest that liver dysfunction without cirrhosis may also be adequate to negatively affect post-surgical outcomes. If more subtle forms of liver dysfunction truly influence cardiac surgical outcomes, their impact is not being reflected by current risk calculator algorithms [6]. The Society of Thoracic Surgery (STS) operative risk calculator acknowledges the impact of liver disease in patients with a known history of hepatitis B, hepatitis C, autoimmune hepatitis, drug-induced hepatitis, cirrhosis, esophageal varices, liver transplant, or congestive hepatopathy, but dismisses a diagnosis of non-alcoholic steatohepatitis (NASH) if cirrhosis is absent [6].

Shear wave elastography identifies silent advanced liver disease in five to seven percent of the general population [7]. The majority of these patients are unaware of having any underlying liver abnormalities [8]. Never-the-less, patients with metabolic disease and even mild increases in liver stiffness often display abnormal myocardial strain [9]. In patients with metabolic disease, elevated liver stiffness is an important predictor of long-term outcomes and all-cause mortality, where cardiovascular disease is a major contributor [9,10,11].

In an exploratory study of patients without typical indicators of liver dysfunction or injury, our group reported an association between preoperative liver stiffness (LSM) and hospital length of stay (LOS) after cardiac surgery [12]. Patients with a LSM ≥ 9.5 kPa stayed on average 3 days longer than those with liver stiffness < 9.5 kPa, and no patients with a LSM ≥ 9.5 kPa had a postoperative length of stay less than 6 days. Such preoperative metrics, which delineate risks within a population previously felt to be homogenous, allow for more accurate risk stratification and may inform the identification of patients unlikely to experience a short postoperative hospital stay. Here, we sought to expand our initial observations by evaluating the predictive ability of liver stiffness to rule out a short length of stay following isolated coronary artery bypass grafting (CABG) surgery.

## 2. Materials and Methods

### 2.1. Study Design

This was a prospective study of 164 patients who underwent isolated CABG. The exposure of interest was liver stiffness (LSM), as measured by ultrasound elastography, which was treated categorically to describe patients with low-to-moderate LSM (<9.5 kPa) versus high LSM (≥9.5 kPa). The primary outcome was hospital length of stay (LOS) < 6 days, with LOS measured in calendar days from the end of surgery to discharge. Thirty-day readmission or death following discharge was evaluated as a secondary outcome.

### 2.2. Study Participants

Adult patients undergoing isolated CABG at a single-center university hospital between December 2019 and March 2023 were eligible for this study. The exclusion criteria included the use of ventricular assist devices prior to surgery and surgical procedures other than isolated CABG. This study was approved by the Institutional Review Board (IRB00225452, 3 December 2019). Written informed consent was obtained from all study participants.

### 2.3. Elastography Assessment

Two operators (GS and JMD) performed all elastography measurements. Both operators received certificates of competence from the manufacturer (Supersonic Imagine, Aix-en-Provence, France) to perform elastography measurements before the start of the study. Liver stiffness was evaluated using two-dimensional shear wave elastography (Aixplorer MACH 30, Supersonic Imagine, Aix-en-Provence, France). The measurements were obtained at baseline before cardiac surgery or on the day of surgery prior to the administration of anesthesia. All patients were without food or drink for greater than four hours before LSM. The patients were in a supine position with the right arm abducted. The measurements were obtained in the right upper lobe of the liver between the seventh and eighth intercostal spaces at end expiration during normal tidal breathing. Five measurements were taken for each subject. The measurements were accepted as valid if they met criteria pre-defined by the manufacturer. The median value of the 5 measurements was accepted if the IQR/median was <30%.

### 2.4. Baseline Covariates

The baseline demographic characteristics, comorbidities, and laboratory data were extracted from the medical record and the Society of Thoracic Surgeons (STS) National Database. Medical care throughout the hospital course was directed by the clinical team. The clinical team was blinded to the results of the liver elastography evaluation. Follow-up included intra- and post-operative care phases until hospital discharge. Complications were determined from the abstraction of data routinely recorded in the medical record.

### 2.5. Outcome Measures

The primary outcome was LOS < 6 days, with LOS measured from the end of surgery to discharge. Thirty-day readmission or death following discharge was evaluated as a secondary outcome.

### 2.6. Statistical Methods

Patient and surgical characteristics were compared between LSM groups (<9.5 kPa vs. ≥9.5 kPa). Continuous variables were summarized as medians and interquartile ranges (IQR) and analyzed using Wilcoxon rank sum. Categorical variables are summarized and analyzed using Chi-square or Fisher’s exact tests, as appropriate. Prior to primary analyses, correlations between LSM, the outcome (LOS), and key covariates (Fibrosis-4 Index (FIB-4) and central venous pressure (CVP)) were assessed descriptively. The sensitivity and specificity of LSM (<9.5 kPa vs. ≥9.5 kPa) to predict LOS < 6 days were also evaluated.

Multivariate logistic regression models were used to assess the adjusted relationship between LSM and a short hospital stay (LOS < 6 days). In addition to LSM, age, sex, STS predicted morbidity/mortality, and baseline hemoglobin were selected a priori for inclusion in multivariate models based on extant literature [13,14,15,16]. A receiver operating characteristic (ROC) curve was generated for the final multivariate model, and the area under the curve (AUC) was estimated to evaluate the discriminatory ability of LSM (≥9.5 vs. <9.5) to predict a hospital LOS < 6 days while controlling for other covariates. Multivariate logistic regression models were also employed to evaluate the adjusted relationship between LSM and the secondary outcome, 30-day readmission or death. Statistical significance was defined as *p* < 0.05. Analyses were performed using SAS 9.4.

## 3. Results

### 3.1. Patient Characteristics

One hundred sixty-six patients who underwent isolated CABG surgery were enrolled in this study. All surgeries were performed using cardiopulmonary bypass, and an aortic cross clamp was used in all but one case. Eighty-four of these patients were analyzed previously as part of a separate study [12]. One patient died prior to discharge and was excluded from the analysis. A separate patient experienced a severe intraoperative complication necessitating a second cardiac surgery and was excluded from the analysis. Both excluded patients had a LSM ≥ 9.5 kPa. In total, 164 patients were analyzed in this study.

The patients in this cohort were predominantly male (84.8%) with normal biventricular and tricuspid valve function who underwent isolated CABG surgery. One hundred forty-one patients (86%) demonstrated low-to-moderate LSM (<9.5 kPa), and 23 patients (14%) demonstrated high LSM (≥9.5 kPa). Compared to patients with low-to-moderate LSM, those with high LSM experienced more sleep apnea (4.3% vs. 26.1%, *p* < 0.01), history of atrial fibrillation (7.1% vs. 30.4%, *p* < 0.01), and higher baseline central ventral pressures (median: 9, interquartile range (IQR): 7–12) vs. median: 10, IQR: 8–15, *p* = 0.01) (Table 1). Notably, groups did not differ with respect to the presence of metabolic syndrome, markers of liver (alcohol consumption, LFTs, FIB-4, Model for End-Stage Liver Disease (MELD) score), kidney (glomerular filtration rate (GFR), baseline creatinine, use of dialysis), or chronic lung disease. The presence of current heart failure or cardiogenic shock and the interval from myocardial infarction to surgery (when applicable) did not differ between groups. Additionally, time on cardiopulmonary bypass and aortic cross clamp times did not differ between groups (Supplementary Table S1).

### 3.2. Hospital Length of Stay

**Figure 1:**
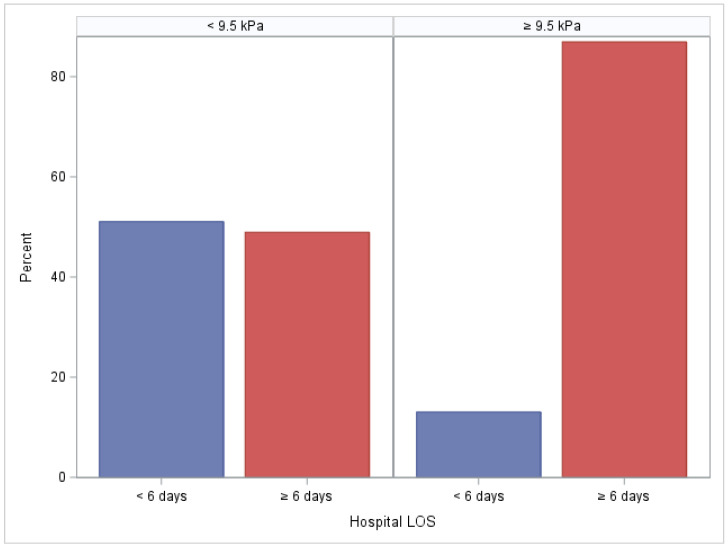
Histogram plot depicting the percent of subjects in each LSM group (LSM < 9.5 kPa and LSM ≥ 9.5 kPa) who had a hospital length of stay less than 6 days and greater than 6 days.

**Figure 2:**
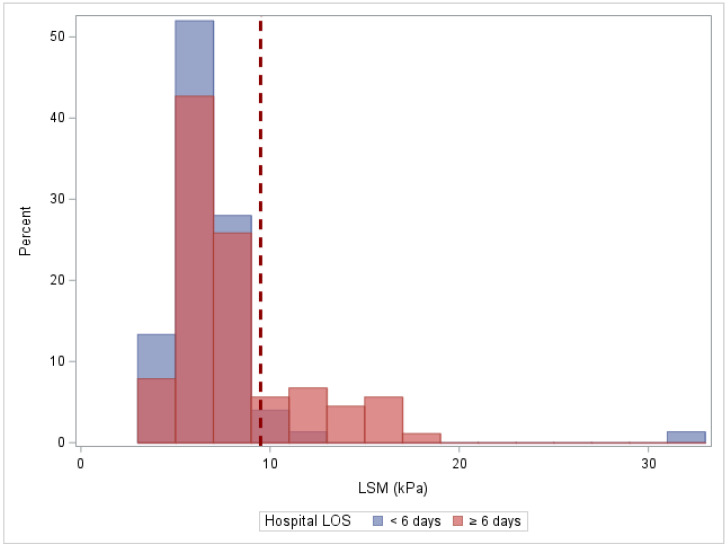
Histogram plot depicting the distribution of liver stiffness by hospital length of stay. The dark shade indicates an overlap of blue and orange coloring. The dashed line identifies an LSM value of 9.5 kPa.

The median hospital length of stay after surgery was 6 days, with a range of 3–35 days. Subjects with high LSM had a higher median length of stay as compared to subjects with low-to-moderate LSM (8 [IQR: 6–10] vs. 5 [IQR: 5–7], respectively, *p* < 0.01). Of the 23 subjects with a LSM ≥ 9.5 kPa, only 3 (13%) had a hospital length of stay less than 6 days. Of the 141 subjects with a LSM < 9.5 kPa, 72 (51%) had a hospital length of stay less than 6 days (*p* < 0.01) (Figure 1). The sensitivity and specificity of LSM ≥ 9.5 to predict a LOS ≥ 6 days were 0.22 (95% CI: 0.14–0.31) and 0.96 (95% CI: 0.92–1), respectively. The positive predictive value of liver stiffness to predict a length of stay ≥ 6 days was 0.87 (95% CI: 0.73–1) (Figure 2). There were no outliers with a short length of stay. There were six outliers with a long length of stay (1.5 × IQR Q3, greater than 12 days).

Univariate models confirmed that the independent variables selected a priori (age (*p* = 0.0084), sex (*p* = 0.003), baseline hemoglobin (*p* = 0.013), LSM (<9.5 kPa, ≥9.5 kPa) (*p* = 0.003), and STS predicted morbidity and mortality (*p* = 0.0004)) were each significantly associated with a LOS < 6 days.

**Figure 3:**
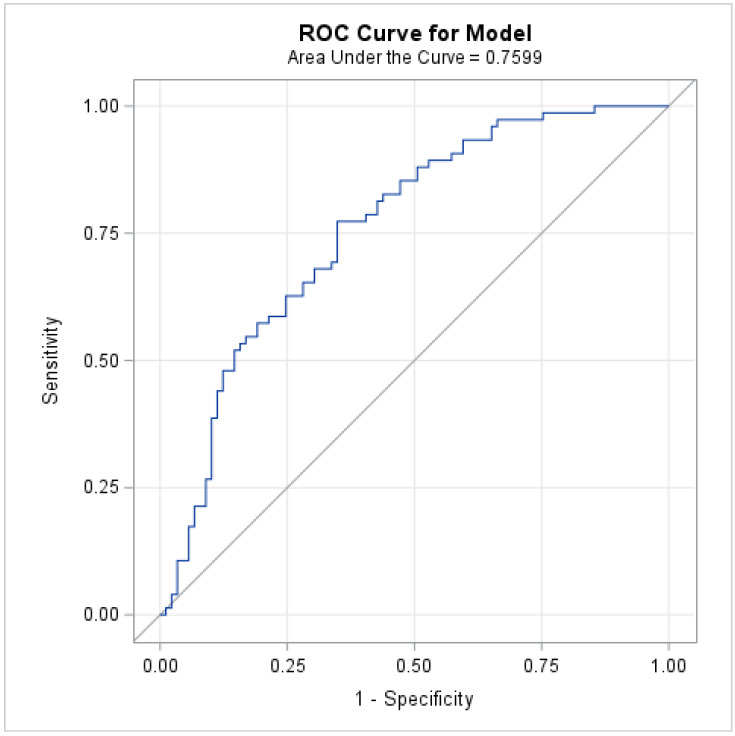
A receiver operating characteristic (ROC) curve was generated for the final multivariate model, and the area under the curve (AUC) was estimated to evaluate the discriminatory ability of LSM (≥9.5 vs. <9.5) to predict a hospital LOS < 6 days, while controlling for other covariates.

We performed multivariate logistic regression models using short hospital LOS (<6 days) as the dependent variable. Independent variables included LSM (<9.5 kPa, ≥9.5 kPa), age, sex, STS predicted morbidity and mortality, and baseline hemoglobin. After adjusting for all variables, high LSM was associated with lower odds of early discharge as compared to low-to-moderate LSM (OR: 0.22, 95% CI: 0.06–0.84, *p* = 0.03). Other covariates associated with lower odds of a short stay included female sex (OR: 0.18, 95% CI: 0.05–0.64) and higher STS predicted morbidity and mortality (OR: <0.01, 95% CI < 0.01–0.37) (Table 2). The ROC curve and resulting AUC of 0.76 (95% CI: 0.68–0.83) suggest the final multivariate model provides acceptable to good discriminatory performance when predicting early discharge (Figure 3).

### 3.3. Readmission or Death within 30 Days Following Discharge

There were 14 patients who experienced hospital readmission or death within 30 days of discharge. Nine of these patients were from the group of 141 with LSM < 9.5 kPa (6.3%). Five of the 14 patients readmitted or died within 30 days of discharge were from the group of 23 who had a LSM ≥ 9.5 kPa (21.7%) (*p* = 0.02). However, a multivariate model adjusting for LSM, age, sex, STS predicted MM, and baseline hemoglobin identified no significant predictors of 30-day readmission or death.

### 3.4. Perioperative Blood Product Administration

When compared to patients with a LSM < 9.5kPa, those with a LSM ≥ 9.5 kPa were more likely to receive PRBCs (*p* = 0.015) and cryoprecipitate (*p* = 0.03). There were no significant differences in the administration of plasma (*p* = 0.07) or platelets (*p* = 0.88) between LSM groups (Supplemental Figure S1).

### 3.5. Discharge Location

There were nine patients discharged to a non-home location. Seven of these patients were from the group of 141 with a LSM < 9.5 kPa (5%) and two patients were from the group of 23 with a LSM ≥ 9.5 kPa (8.7%). The association between discharge location and LSM after isolated CABG surgery was not statistically significant (*p* = 0.62).

### 3.6. Postoperative Infection

There were 17 patients who had a postoperative infection. Eleven of these patients were from the group of 141 with a LSM < 9.5 kPa (7.5%), and six patients were from the group of 23 with a LSM > 9.5 kPa (26%). The association between postoperative infection and LSM was statistically significant (*p* = 0.017) (Table 3).

## 4. Discussion

This prospective observational study evaluated whether preoperative liver stiffness could identify patients unlikely to exit the hospital within the first 6 days following isolated CABG surgery. We found that, after adjusting for age, gender, baseline hemoglobin, and an illness severity score (the STS morbidity and mortality risk), patients with a preoperative liver stiffness greater than or equal to 9.5 kPa as assessed by 2D shear wave elastography were much less likely to be discharged from the hospital within 6 days of isolated CABG surgery when compared to those with a LSM less than 9.5 kPa. The odds ratio of an early discharge for patients with a liver stiffness greater than 9.5 kPa was 0.22, suggesting that nearly 80% of patients with elevated liver stiffness will not have an early discharge following isolated CABG surgery. Though higher LSM is associated with 30-day readmission/death, it does not predict these outcomes in a multivariate analysis. The implications of these findings are twofold: that liver stiffness may provide useful supplementary data for identifying the best candidates for fast tracking after isolated CABG surgery, and that liver stiffness may serve as a measure to gauge preoperative optimization prior to elective isolated CABG surgery.

Processes known to influence liver stiffness include organic liver disease resulting in fibrosis, cholestatic disease, infiltrative liver disease, central venous pressure, and inflammation [17]. Although an exhaustive evaluation of the relative contribution of each of these processes was beyond the scope of this study, we did assess the correlation between liver stiffness and the FIB-4 index (a liver fibrosis biomarker that is intended to aid in the early diagnosis of advanced liver disease) [18,19], as well as the correlation between liver stiffness and CVP. We found no significant correlation between liver stiffness and the FIB-4 index (*p* = 0.06). Though a significant correlation between CVP and liver stiffness was observed (*p* = 0.03), the correlation coefficient was low (r = 0.18). This suggests that CVP was not likely to be a dominant driver of liver stiffness in this population. The presence of metabolic disease and alcohol consumption are known to be associated with liver stiffness in the general population [20]. In our current study, the prevalence of neither metabolic syndrome nor reported alcohol consumption differed between high- and low-stiffness groups.

In our prior study, the failure of no single organ system could be identified as commonly responsible for the delayed discharge in patients with elevated baseline liver stiffness [12]. This is consistent with the nonspecific character of the pathophysiology reflected by liver stiffness and suggests that identifying unlikely candidates for early discharge may be easier than converting these patients into early discharge candidates. It is possible that liver stiffness is one reflection of how an individual patient responds to these risk factors. That is, some patients who have metabolic syndrome or its individual components may have more resilience and maintain a normal liver stiffness, while other patients may be more susceptible to these systemic processes, which manifest as elevated liver stiffness.

We did, however, identify in the current study three additional outcomes more commonly observed in patients with elevated baseline liver stiffness: greater perioperative blood product administration, a higher incidence of postoperative infection, and a higher rate of 30-day readmission or death. It is possible that the silent liver disease present in the high stiffness cohort predisposes to a suboptimal response to major surgical stress, resulting in greater perioperative morbidity.

Patients with documented cirrhosis make up less than 1% of patients in the STS database [21]. By contrast, patients with a MELD score of 9 or greater (suggesting at least mild liver dysfunction) make up roughly 25% of the STS population [22]. Thus, an understanding of the impact of non-cirrhotic liver disease on cardiac surgery patients may impact a greater percentage of patients than does a focus on cirrhotic liver disease. Notably, in our study, we did not observe a significant difference in MELD scores between the high and low stiffness groups. This suggests that ultrasound elastography may be more sensitive for detecting subclinical liver damage and may be more useful as a preoperative screening tool.

The strengths of this study include its prospective design, the homogeneity of surgical procedures performed, and the blinding of the treatment teams to the elastography results. The limitations of this study include a lack of liver biopsy data and a lack of formal assessment of the presence of fatty liver disease. We used a cut-off value of 9.5 kPa to identify the high stiffness group, which was consistent with several other studies and our previous report. Future studies may explore alternative liver stiffness values to determine the optimal cut-point for risk stratification and may explore whether interventions targeted at decreasing liver stiffness prior to isolated CABG surgery increase the probability of early discharge following isolated CABG surgery.

## 5. Conclusions

In 164 patients undergoing isolated CABG, a preoperative LSM ≥ 9.5 kPa ruled out a short length of stay in 80% of patients when compared to patients with a LSM < 9.5 kPa. Preoperative liver stiffness appears to be a useful marker for identifying patients who are unlikely to have an early discharge after isolated CABG surgery and may be a useful metric to incorporate into preoperative risk models.

## Figures and Tables

**Table 1:** Baseline patient characteristics.

	LSM < 9.5 kPa	LSM > 9.5 kPa	Total	*p*
N (%)	141 (85.98)	23 (14.02)	164 (100)	
LSM (kPa) (median [IQR])	6.3 (5.6–7.5)	12 (11–15.4)	6.65 (5.7–8.15)	0.000
Sex				
Male	121 (85.82)	18 (78.26)	139 (84.76)	0.350
Female	20 (14.18)	5 (21.74)	25 (15.24)	
Age (years) (median [IQR])	63 (57–69)	68 (61–75)	63 (57.5–70)	0.077
BMI (median [IQR])	28 (26.14–30.62)	27.8 (25.77–32.4)	27.99 (26.09–30.89)	0.516
Prior Cardiac Procedure (PCI/AICD/Ablation)				
No	107 (75.89)	17 (73.91)	124 (75.61)	0.838
Yes	34 (24.11)	6 (26.09)	40 (24.39)	
Diabetes				
No	80 (56.74)	12 (52.17)	92 (56.1)	0.683
Yes	61 (43.26)	11 (47.83)	72 (43.9)	
HgbA1c (baseline) (median [IQR])	6.1 (5.5–7.1)	6.2 (5.7–7.5)	6.1 (5.6–7.3)	0.576
TGL (median [IQR])	125 (88–173)	147 (71.5–198)	126 (85–175)	0.968
HDL (median [IQR])	41 (35–49)	40.5 (33.5–49.5)	41 (34–49)	0.932
Hypertension				
No	23 (16.31)	3 (13.04)	26 (15.85)	1.000
Yes	118 (83.69)	20 (86.96)	138 (84.15)	
Metabolic Syndrome				
No	75 (53.19)	11 (47.83)	86 (52.44)	0.633
Yes	66 (46.81)	12 (52.17)	78 (47.56)	
Known Liver Disease				
No	136 (96.45)	21 (91.3)	157 (95.73)	0.255
Yes	5 (3.55)	2 (8.7)	7 (4.27)	
Hgb (baseline) (median [IQR])	13.9 (12.8–15.1)	13.2 (11–14.1)	13.85 (12.75–15)	0.026
Diagnosed OSA (CPAP)				
No	135 (95.74)	17 (73.91)	152 (92.68)	0.002
Yes	6 (4.26)	6 (26.09)	12 (7.32)	
Atrial Fibrillation Hx				
No	131 (92.91)	16 (69.57)	147 (89.63)	0.003
Yes	10 (7.09)	7 (30.43)	17 (10.37)	
Dialysis				
No	139 (98.58)	23 (100)	162 (98.78)	1.000
Yes	2 (1.42)	0 (0)	2 (1.22)	
Creatinine (baseline) (median [IQR])	1 (0.9–1.1)	1 (0.8–1.3)	1 (0.9–1.2)	0.662
GFR (baseline) (median [IQR])	77 (66–90)	76 (56–86)	77 (66–90)	0.460
LVEF (avg) (median [IQR])	57.5 (52.5–62.5)	57.5 (42.5–62.5)	57.5 (52.5–62.5)	0.845
LVEDd (dim) (median [IQR])	45.5 (41.1–50.9)	44 (41.4–53.2)	45.45 (41.25–51)	0.623
CVP (median [IQR])	9 (7–12)	10 (8–15)	9 (7–12)	0.014
RV Function				
Normal	133 (94.33)	19 (82.61)	152 (92.68)	0.068
Abnormal	8 (5.67)	4 (17.39)	12 (7.32)	
Tricuspid Regurgitation				
None	24 (17.02)	5 (21.74)	29 (17.68)	0.530
Trace	101 (71.63)	14 (60.87)	115 (70.12)	
Mild	14 (9.93)	4 (17.39)	18 (10.98)	
Moderate	2 (1.42)	0 (0)	2 (1.22)	
Chronic Lung Disease				
No	126 (89.36)	17 (73.91)	143 (87.2)	0.084
Yes	15 (10.64)	6 (26.09)	21 (12.8)	
IV Drug Abuse				
Unknown	1 (0.71)	0 (0)	1 (0.61)	1.000
No	136 (96.45)	23 (100)	159 (96.95)	
Yes	4 (2.84)	0 (0)	4 (2.44)	
Alcohol use (drinks per week)				
Unknown	1 (0.71)	0 (0)	1 (0.61)	0.249
None	77 (54.61)	13 (56.52)	90 (54.88)	
≤1	28 (19.86)	6 (26.09)	34 (20.73)	
2–7	27 (19.15)	1 (4.35)	28 (17.07)	
≥8	8 (5.67)	3 (13.04)	11 (6.71)	
FIB4 (median [IQR])	1.31 (1.02–1.96)	1.59 (1.07–2.57)	1.33 (1.03–2.05)	0.217
Albumin (baseline) (median [IQR])	4.3 (4.1–4.6)	4.2 (3.7–4.5)	4.3 (4–4.6)	0.075
ALT (baseline) (median [IQR])	27 (19–33.5)	26 (18–35)	27 (19–34)	0.812
AST (baseline) (median [IQR])	24 (19–32)	23 (18–28)	23.5 (18.5–31)	0.841
Bilirubin (baseline) (median [IQR])	0.5 (0.3–0.7)	0.6 (0.4–0.8)	0.5 (0.4–0.7)	0.295
Plt (baseline) (median [IQR])	216,000 (184,000–266,000)	209,000 (159,000–267,000)	215,500 (182,000–266,000)	0.394
MELD score (median [IQR])	6 (6–7)	6 (6–8)	6 (6–7)	0.976

Abbreviations: LSM: Liver Stiffness Measurement; BMI: Body Mass Index; HgbA1c: Hemoglobin A1C; TGL: Triglycerides; HDL: High Density Lipoprotein; Hgb: Hemoglobin; OSA: Obstructive Sleep Apnea; GFR: Glomerular Filtration Rate; LVEF: Left Ventricular Ejection Fraction; LVEDd: Left Ventricular End Diastolic Dimension; CVP: Central Venous Pressure; RV: Right Ventricular; ALT: Alanine Aminotransferase; AST: Aspartate Aminotransferase; Plt: Platelet; MELD: Model for End Stage Liver Disease; PCI: Percutaneous Coronary Intervention; AICD: Automatic Implantable Cardioverter-Defibrilator.

**Table 2:** Univariate and multivariate analyses for each predictor of a short length of stay (selected a priori) and their *p* value.

	Univariate		Multivariate	
Variable	Odds Ratio (95% CI)	*p*	Odds Ratio (95% CI)	*p*
LSM kPa (≥9.5 vs. <9.5)	0.144 (0.041–0.506)	0.0025	0.224 (0.059–0.843)	0.027
Age	0.955 (0.923–0.988)	0.0084	0.961 (0.924–0.999)	0.045
STS Predicted Morbidity and Mortality	<0.001 (<0.001–<0.001)	0.0004	<0.001 (<0.001–0.372)	0.031
Sex (Female vs. Male)	0.182 (0.060–0.559)	0.003	0.179 (0.05–0.64)	0.008
Baseline Hemoglobin	1.248 (1.049–1.486)	0.0126	0.958 (0.769–1.193)	0.700

**Table 3:** Secondary outcomes.

	LSM < 9.5 kPa	LSM ≥ 9.5 kPa	Total	*p*
N (%)	141 (85.98)	23 (14.02)	164 (100)	
Post Op Atrial Fibrillation				
No	98 (69.5)	12 (52.17)	110 (67.07)	0.101
Yes	43 (30.5)	11 (47.83)	54 (32.93)	
Discharge Location Code				
Home	134 (95.04)	21 (91.3)	155 (94.51)	0.615
Non-home	7 (4.96)	2 (8.7)	9 (5.49)	
Intraoperative Blood Products Administered				
No	98 (69.5)	13 (56.52)	111 (67.68)	0.217
Yes	43 (30.5)	10 (43.48)	53 (32.32)	
Postoperative Blood Products Administered				
No	92 (65.25)	9 (39.13)	101 (61.59)	0.017
Yes	49 (34.75)	14 (60.87)	63 (38.41)	
Total Cryoprecipitate (units) (median [IQR])	0 (0–0)	0 (0–1)	0 (0–0)	0.031
Total FFP (units) (median [IQR])	0 (0–0)	0 (0–0)	0 (0–0)	0.068
Total PLT (units) (median [IQR])	0 (0–1)	0 (0–1)	0 (0–1)	0.881
Total RBC (units) (median [IQR])	0 (0–1)	1 (0–5)	0 (0–2)	0.015
Hospital LOS (days) (median [IQR])	5 (5–7)	8 (6–10)	6 (5–8)	0.001
ICU LOS (days) (median [IQR])	1.57 (0.96–2.8)	1.85 (0.99–4.77)	1.59 (0.96–2.9)	0.111
ICU LOS (hours) (median [IQR])	37.6 (23–67.18)	44.3 (23.8–114.5)	38.07 (23.09–69.52)	0.111
Hospital LOS categorical				
<6 days	72 (51.06)	3 (13.04)	75 (45.73)	0.001
≥6 days	69 (48.94)	20 (86.96)	89 (54.27)	
30-day readmission or death				
Unknown	1 (0.71)	1 (4.35)	2 (1.22)	0.018
No	131 (92.91)	17 (73.91)	148 (90.24)	
Yes	9 (6.38)	5 (21.74)	14 (8.54)	
Postoperative infection or death				
No	130 (92.2)	17 (73.91)	147 (89.63)	0.017
Yes	11 (7.8)	6 (26.09)	17 (10.37)	
Total ventilator days (median [IQR])	1 (0–1)	1 (0–1)	1 (0–1)	0.592

Abbreviations: FFP—Fresh Frozen Plasma; PLT—Platelets; RBC—Red Blood Cells; LOS—Length of Stay; ICU—Intensive Care Unit.

## Data Availability

The original contributions presented in the study are included in the article/Supplementary Material; further inquiries can be directed to the corresponding author.

## Supplementary Materials

The following supporting information can be downloaded at: https://www.mdpi.com/article/10.3390/jcm13123397/s1, Supplemental Figure S1: Box and whisker plot depicting the total units of blood products transfused during the perioperative period for subjects with LSM < 9.5 kPa and ≥9.5 kPa. Supplementary Table S1: Additional baseline patient characteristics.
