# Supplementary material for: The Impact of Silent Liver Disease on Hospital Length of Stay Following Isolated Coronary Artery Bypass Grafting Surgery
Source: J Clin Med. 2024 Jun 10;13(12):3397. doi: 10.3390/jcm13123397 (PMC11204604; doi:10.3390/jcm13123397)
Supplement: Supplementary file 1 [file jcm-13-03397-s001.zip › jcm-2997388-supplementary.pdf]

Supplemental Figure S1

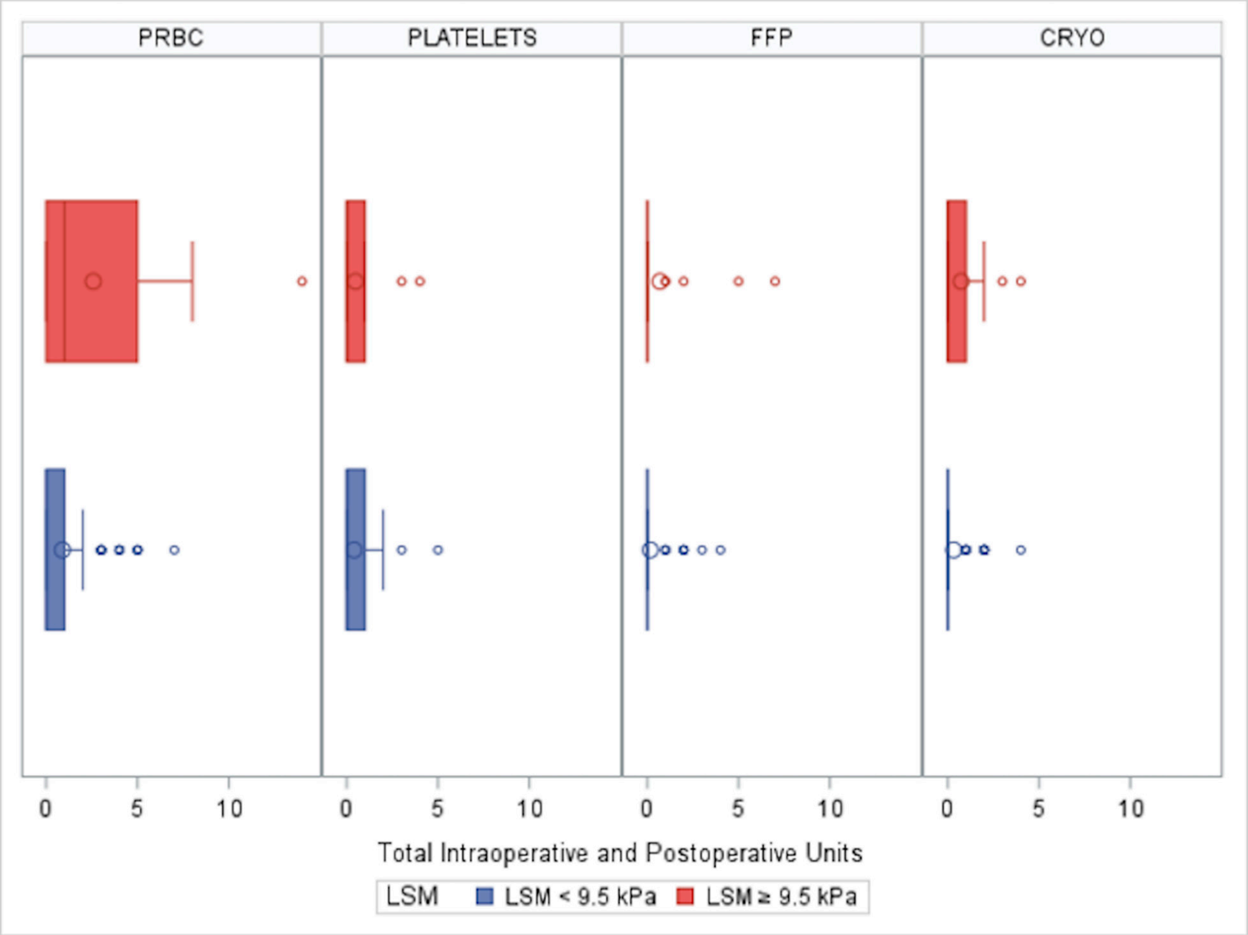

Supplemental Table S1

| | LSM < 9.5 kPa | LSM ≥ 9.5 kPa | Total | |
|---|---|---|---|---|
| | ***n* (%)** | ***n* (%)** | ***n* (%)** | ***p*** |
| ***n*** | 141 (85.98) | 23 (14.02) | 164 (100) | |
| **Heart Failure Timing** | | | | |
| **Unknown** | 6 (4.26) | 0 (0) | 6 (3.66) | 0.834 |
| **None** | 112 (79.43) | 19 (82.61) | 131 (79.88) | |
| **Acute** | 2 (1.42) | 0 (0) | 2 (1.22) | |
| **Chronic** | 12 (8.51) | 3 (13.04) | 15 (9.15) | |
| **Both** | 9 (6.38) | 1 (4.35) | 10 (6.1) | |
| **Cardiogenic Shock** | | | | |
| **No** | 139 (98.58) | 22 (95.65) | 161 (98.17) | 0.366 |
| **Yes** | 2 (1.42) | 1 (4.35) | 3 (1.83) | |
| **Resuscitation** | | | | |
| **No** | 140 (99.29) | 23 (100) | 163 (99.39) | 1.000 |
| **Yes** | 1 (0.71) | 0 (0) | 1 (0.61) | |
| **Time from MI to Surgery** | | | | |
| **Unknown** | 74 (52.48) | 11 (47.83) | 85 (51.83) | 0.899 |
| **< 1 day** | 1 (0.71) | 0 (0) | 1 (0.61) | |
| **1–7 days** | 29 (20.57) | 6 (26.09) | 35 (21.34) | |
| **8–21 days** | 16 (11.35) | 2 (8.7) | 18 (10.98) | |
| **> 21 days** | 21 (14.89) | 4 (17.39) | 25 (15.24) | |
| **Cardiopulmonary Bypass Time (min) (median [IQR])** | 98 (76 – 127) | 103 (62 – 150) | 98.5 (75.5 – 131.5) | 0.865 |
| **Aortic Cross Clamp Time (min) (median [IQR])** | 79 (64 – 103) | 74 (50 – 113) | 77 (59 – 103) | 0.399 |
| **STS Predicted Morbidity and Mortality (median [IQR])** | 0.06 (0.04 – 0.08) | 0.12 (0.06 – 0.15) | 0.06 (0.04 – 0.1) | **0.001** |
| **STS Predicted Mortality (median [IQR])** | 0.01 (0 – 0.01) | 0.01 (0.01 – 0.02) | 0.01 (0 – 0.01) | **0.002** |
| **STS Probability of Long LOS (median [IQR])** | 0.02 (0.01 – 0.03) | 0.04 (0.03 – 0.06) | 0.02 (0.01 – 0.04) | **0.000** |
| **STS Probability of Short LOS (median [IQR])** | 0.63 (0.48 – 0.71) | 0.42 (0.36 – 0.53) | 0.6 (0.45 – 0.7) | **0.000** |
